# Incidence and implications of anti-rituximab antibodies in nephropathies and other immune-mediated diseases: a narrative review

**DOI:** 10.3389/fimmu.2026.1896560

**Published:** 2026-09-15

**Authors:** Marco Allinovi, Jacopo Lomi, Matteo Accinno, Maxime Teisseyre, Michael Diomaiuti, Raffaele Madeo, Federica Allegretta, Andrea Matucci, Alessandra Vultaggio

**Affiliations:** 1 Nephrology, Dialysis and Transplantation Unit, Careggi University Hospital, Florence, Italy; 2 SOC Nefrologia e Dialisi, Ospedale Santa Maria Annunziata di Firenze, Firenze, Italy; 3 Department of Experimental and Clinical Medicine, University of Florence, Florence, Italy; 4 French Reference Center for Rare Diseases, Idiopathic Nephrotic Syndrome and Membranous Nephropathy, Nice University Hospital, Université Côte d’Azur, Nice, France; 5 Immunology Laboratory, Nice University Hospital, Université Côte d’Azur, Nice, France; 6 Institute for Research on Cancer and Aging (IRCAN), Université Côte d’Azur, Nice, France; 7 Unit of Nephrology, San Jacopo Hospital, Pistoia, Italy; 8 Immunoallergology Unit, Careggi University Hospital, Florence, Italy

**Keywords:** anti-drug antibodies, anti-rituximab antibodies, drug desensitization, immunogenicity, membranous nephropathy, rituximab, systemic lupus erythematosus, treatment resistance

## Abstract

Rituximab is a chimeric anti-CD20 monoclonal antibody widely used in immune-mediated and hematologic diseases, but its efficacy may be limited by the development of anti-rituximab antibodies (ADAs). This narrative review summarizes current evidence on the incidence, detection, and clinical implications of ADAs in nephropathies and other immune-mediated disorders. ADA prevalence varies markedly across diseases and is influenced by assay methodology, timing of testing, and patient immune background. Although not all ADAs are clinically relevant, their presence has been associated with lower rituximab levels, earlier B-cell repopulation, reduced clinical/immunological remission, more frequent relapses, and hypersensitivity reactions. Overall, ADAs represent an underrecognized factor that may affect rituximab efficacy and safety, supporting the need for improved monitoring and more personalized therapeutic strategies.

## 1 Introduction

Rituximab is a chimeric monoclonal antibody (mAb) that specifically recognizes CD20 on immature, naïve, mature, and memory human B cells. Composed of an anti-human murine variable region and human constant regions of the IgG1 type, rituximab exploits its specificity for CD20 to selectively bind to B cells, activating immune mechanisms that lead to their depletion (1).

The main mechanisms by which rituximab induces B-cell depletion include complement-mediated cytotoxicity (CDC), antibody-dependent cell-mediated cytotoxicity (ADCC), antibody-dependent cellular phagocytosis (ADCP), and induction of apoptosis via binding to CD20 on the cell membrane (2–7). These mechanisms have made rituximab a key drug in the treatment of several hematologic and autoimmune diseases, including non-Hodgkin’s lymphomas, leukemias, and systemic lupus erythematosus (SLE).

Like other mAbs, rituximab can be immunogenic, stimulating the production of anti-rituximab antibodies, also known as anti-drug antibodies (ADAs) or human anti-chimeric antibodies (HACAs) (8) (**Figure 1**). ADAs can be neutralizing, directly interfering with rituximab’s ability to bind to CD20, or non-neutralizing, acting through other mechanisms, specifically by increasing drug clearance (9). It has been hypothesized that rituximab may be more immunogenic in some autoimmune conditions, such as SLE, where the underlying activation of the immune system and its high reactivity increases the likelihood of ADA formation (10, 11). From a clinical point of view, the production of ADAs may impair therapeutic efficacy (12–19) and/or increase the risk of hypersensitivity reactions (HSRs); although they are generally mild to moderate, fatal outcomes have been reported. These reactions are not specific to rituximab (20) (**Figure 2**).

**Figure 1:**
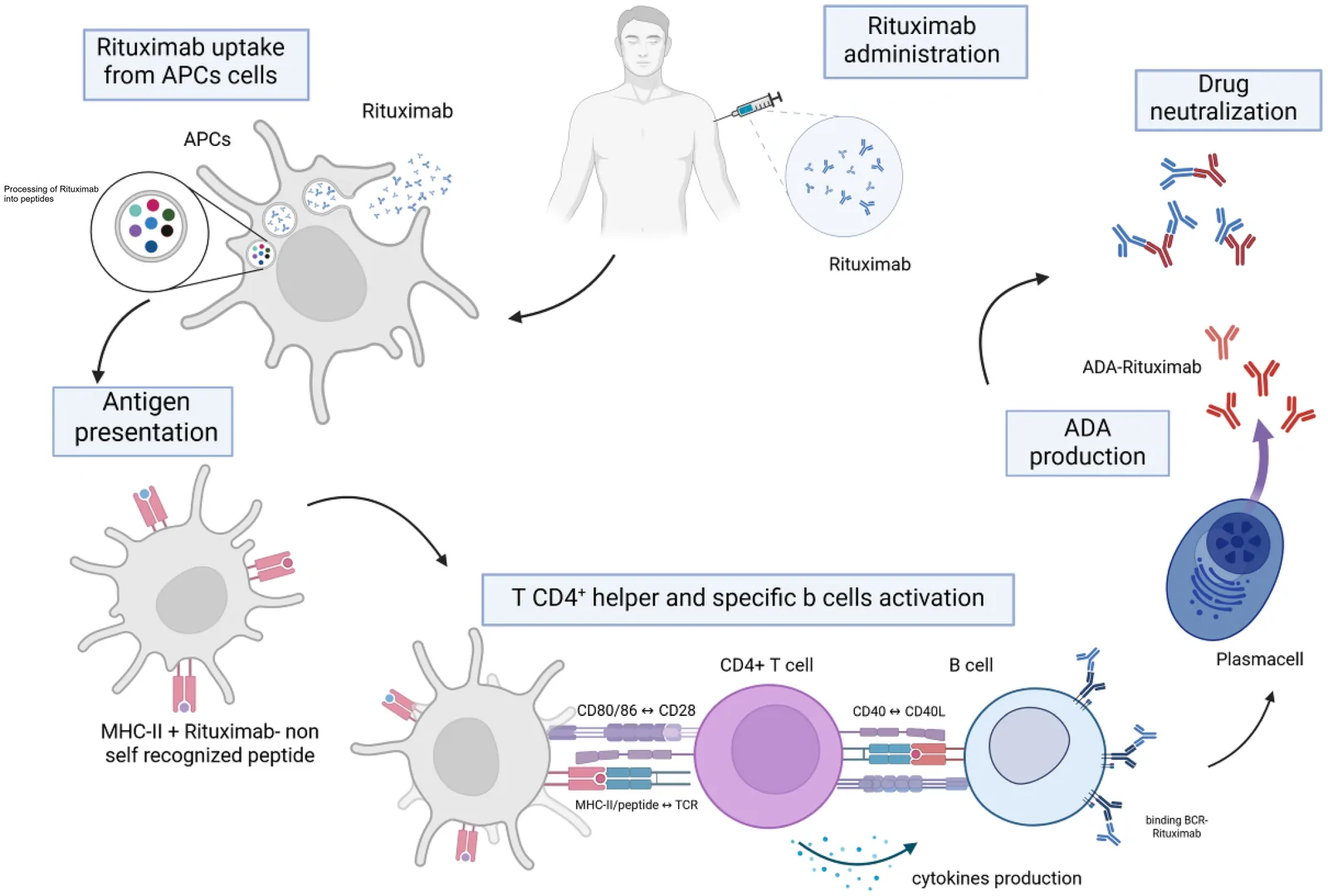
Schematic representation of anti-drug antibody (ADA) development against rituximab. After administration, rituximab was uptaken by antigen-presenting cells (APCs), processed, and presented via MHC-II molecules as non-self peptides. This leads to the activation of CD4^+^ T helper cells through TCR and co-stimulatory signals (CD80/86–CD28). The activated T cells promote antigen-specific B-cell activation via CD40–CD40L interaction and cytokine production. B cells recognize rituximab through their B-cell receptor, leading to ADA production. These anti-rituximab antibodies can bind and neutralize the drug, potentially reducing its therapeutic efficacy.

**Figure 2:**
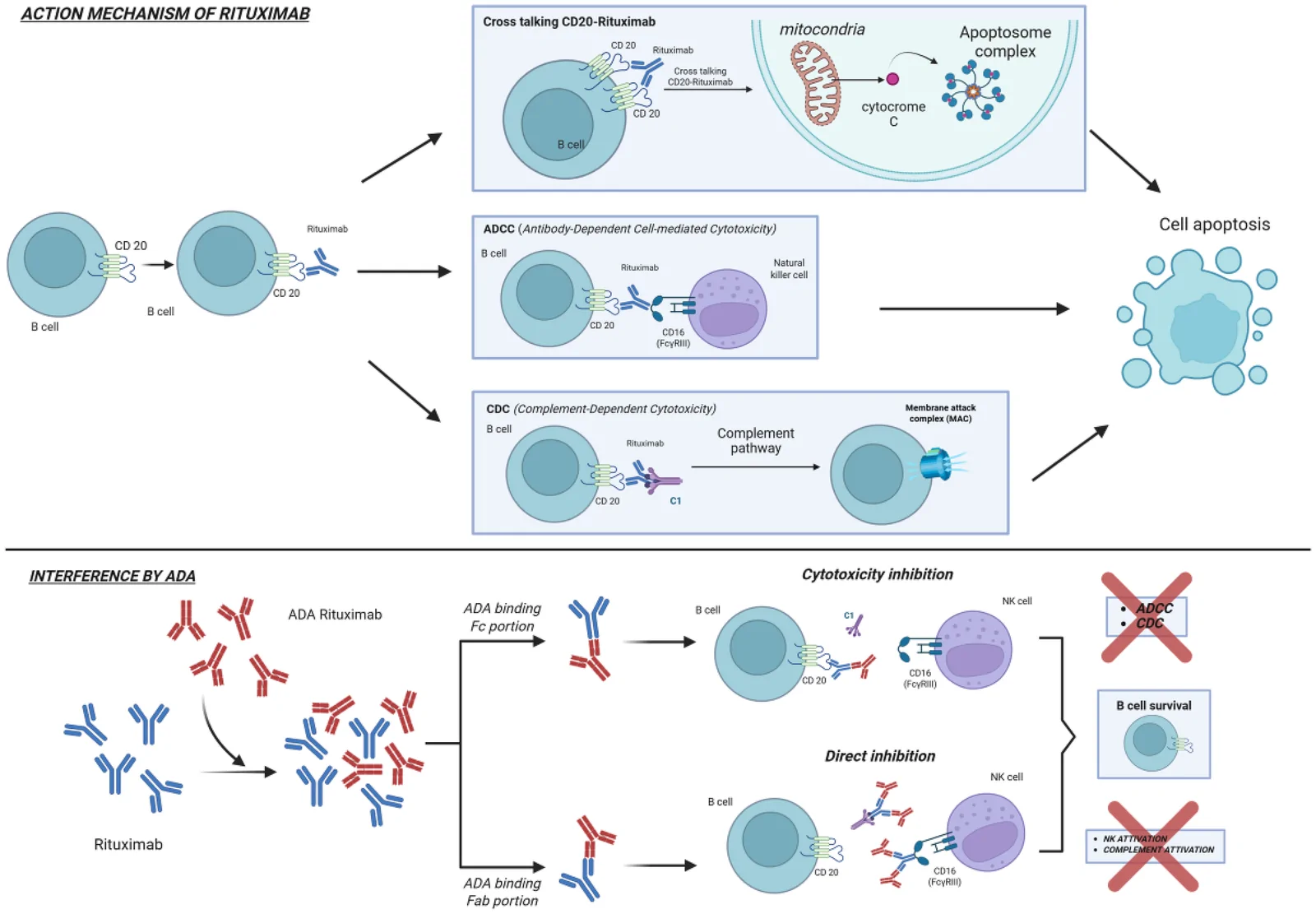
Schematic representation of rituximab mechanisms of action and interference by anti-drug antibodies (ADA). Rituximab binds CD20 on B cells, inducing cell death through multiple mechanisms: (1) direct signaling via CD20 cross-linking leading to mitochondrial apoptosis and apoptosome formation, (2) antibody-dependent cell-mediated cytotoxicity (ADCC) mediated by Fcγ receptor-expressing effector cells such as natural killer cells, and (3) complement-dependent cytotoxicity (CDC) via activation of the complement cascade and membrane attack complex formation. ADAs interfere with rituximab activity through different mechanisms. Binding to the Fc portion impairs effector functions such as ADCC and CDC, reducing cytotoxicity; binding to the Fab portion directly blocks rituximab interaction with CD20 instead, preventing B-cell targeting. These mechanisms ultimately result in reduced drug efficacy and increased B-cell survival.

Despite growing evidence, the routine assay of ADAs is not yet widespread in clinical practice. Monitoring of ADAs could instead offer valuable support in therapeutic management, especially to identify patients who could benefit from a switch to more recent anti-CD20 antibodies, such as ofatumumab or obinutuzumab, which are respectively fully human and humanized mAbs (19, 21–25).

An in-depth understanding of the mechanisms of ADA formation and their clinical impact is key to optimizing the therapeutic use of rituximab and improving personalized patient management.

## 2 Aims

This review aimed to summarize the main findings of studies analyzing the prevalence of ADAs and their clinical implications in patients treated with rituximab to help clinicians navigate this topic in clinical practice.

## 3 Methods

Given the breadth and heterogeneity of the available evidence on the immunogenicity of rituximab, we conducted a narrative review of the literature. This approach was chosen to provide a comprehensive and clinically oriented overview of the topic rather than to perform a quantitative synthesis or a formal meta-analysis.

To identify relevant publications, we performed a comprehensive literature search of the PubMed and Google Scholar databases, covering the period from the initial approval of rituximab up to April 2026. The search strategy was built around the key term “rituximab” in combination with terms indicating an immune response against the drug, such as “anti-rituximab antibodies”, “anti-drug antibodies”, or “human anti-chimeric antibodies”. We deliberately excluded studies not published in English, as well as case reports, expert opinions, and ongoing trials, to focus on original research providing robust clinical or mechanistic data. Additionally, we screened the reference lists of the retrieved articles and relevant reviews to capture any further studies that might have been missed by the primary search. Two investigators (JL and MA) independently screened all identified papers for inclusion based on the study title and abstract. Through this iterative process, we selected a core set of 56 original studies published between 1997 and 2026, which form the evidentiary basis of this review. The selection was guided by the aim of illustrating the key themes of this narrative review, namely: the epidemiology of anti-rituximab antibodies across different diseases, their detection methods, their impact on pharmacokinetics and pharmacodynamics, and their clinical consequences, including treatment failure and hypersensitivity reactions.

## 4 Results

### 4.1 Detection of anti-rituximab antibodies

#### 4.1.1 Analytical methods adopted

Several methods have been employed for the detection of anti-rituximab antibodies (ADAs), each with specific characteristics in terms of sensitivity, specificity, and susceptibility to drug interference (**Table 1**).

**Table 1:** Detection methods of anti-rituximab antibodies and rituximab levels.

Method	References	Matrix	Type of analysis	Unit of measurement	Positivity cutoff
ELISA (bridging)	(19) (26) (27) (28) (29)	Serum/plasma	ADA	ng/mL	5 ng/mL
ELISA (sandwich)	(19) (26) (28) (11) (30)	Serum/plasma	Rituximab	µg/mL	2 µg/mL 2 µg/mL 2 µg/mL 0.3 μg/ml 0.1 μg/ml
ECL (bridging)	(11) (18) (13) (11)	Serum/plasma	ADA	AU/mL	Internally defined (1% false positive rate)
ECL (sandwich)	(31)	Serum/plasma	Rituximab	ng/mL	20 ng/mL

Detection methods of anti-rituximab antibodies (ADAs) and rituximab levels with the positivity cutoffs for each assay and each study.

ADA, anti-rituximab antibodies; AU, arbitrary units; ECL, electrochemiluminescence; RU, resonance unit.

##### 4.1.1.1 Enzyme-linked immunosorbent assay

It is the most widely used method in the literature, especially in older studies or in contexts with limited resources (**Table 2**) (19, 26–28). It is based on the immobilization of rituximab on a plate; if ADAs are present in the serum, these form a “bridge” between the rituximab on the plate and the labeled one. After washing, the addition of the substrate leads to the development of a colorimetric signal proportional to the amount of ADAs present (31). The advantages include ease of use and relatively low cost; a possible disadvantage is limited sensitivity (due to interference from circulating rituximab), with risk of false negatives (**Figure 3**).

**Table 2:** Prevalence of ADAs in different diseases.

Renal diseases/systemic diseases with recognized renal involvement				
ADA prevalence	Disease	Predominant assay method	Typical sampling timepoints post-rituximab	References
11%–47%	Primary membranous nephropathy	ELISA	3, 6, 9, 12 months	(16, 19, 32–35).
15.1%–74%	Systemic lupus erythematosus	ELISA, ECL	1, 3, 6, 12, 36 months	(11, 12, 15, 18, 23, 27, 36–40)
27%	Sjögren’s syndrome	ELISA	6 months	(41).
0%–25%	ANCA-associated vasculitis	ELISA, ECL	6 months	(18, 35, 37).
0%	Acute pediatric kidney transplant rejection	ELISA	Variable (post-transplant)	(42)
10.5%–48.5%	Childhood-onset frequent-relapsing or steroid-dependent nephrotic syndrome	ELISA, ECL	1, 6, variable months	(14, 26, 43–47)

Diseases without characteristic renal involvement				
ADA prevalence	Disease	Predominant assay method	Typical sampling timepoints post-rituximab	References
37.6%	Myasthenia gravis	ELISA	12 months	(48)
11.7%–36.9%	Neuromyelitis optica spectrum disorder	ELISA	3, 6, 9, 12 months up to 5 years	(49, 50)
24.6%–37%	Relapsing–remitting multiple sclerosis	ELISA, ECL	3, 6, 12, variable months	(13, 51, 52)
7.5%–26%	Primary progressive multiple sclerosis	ELISA, ECL	3, 6 up to 24 months	(13, 53)
18.2%–31%	Pemphigus	ELISA	12, up to 36 months	(17, 54)
0%–20.9%	Lymphoma	ELISA	2, variable months	(8, 31, 55–58)
0%	Myalgic encephalopathy/chronic fatigue syndrome	ECL	3, 6, 10, 15 months	(59)
0.9%–51.6%	Rheumatoid arthritis	ELISA, ECL	3, 6 months (after each course) up to 36 months	(22, 28, 30, 60–69)

ANCA, anti neutrophil cytoplasmic antibodies; ADA, anti-rituximab antibodies; ELISA, enzyme-linked immunosorbent assay; ECL, electrochemiluminescence.

**Figure 3:**
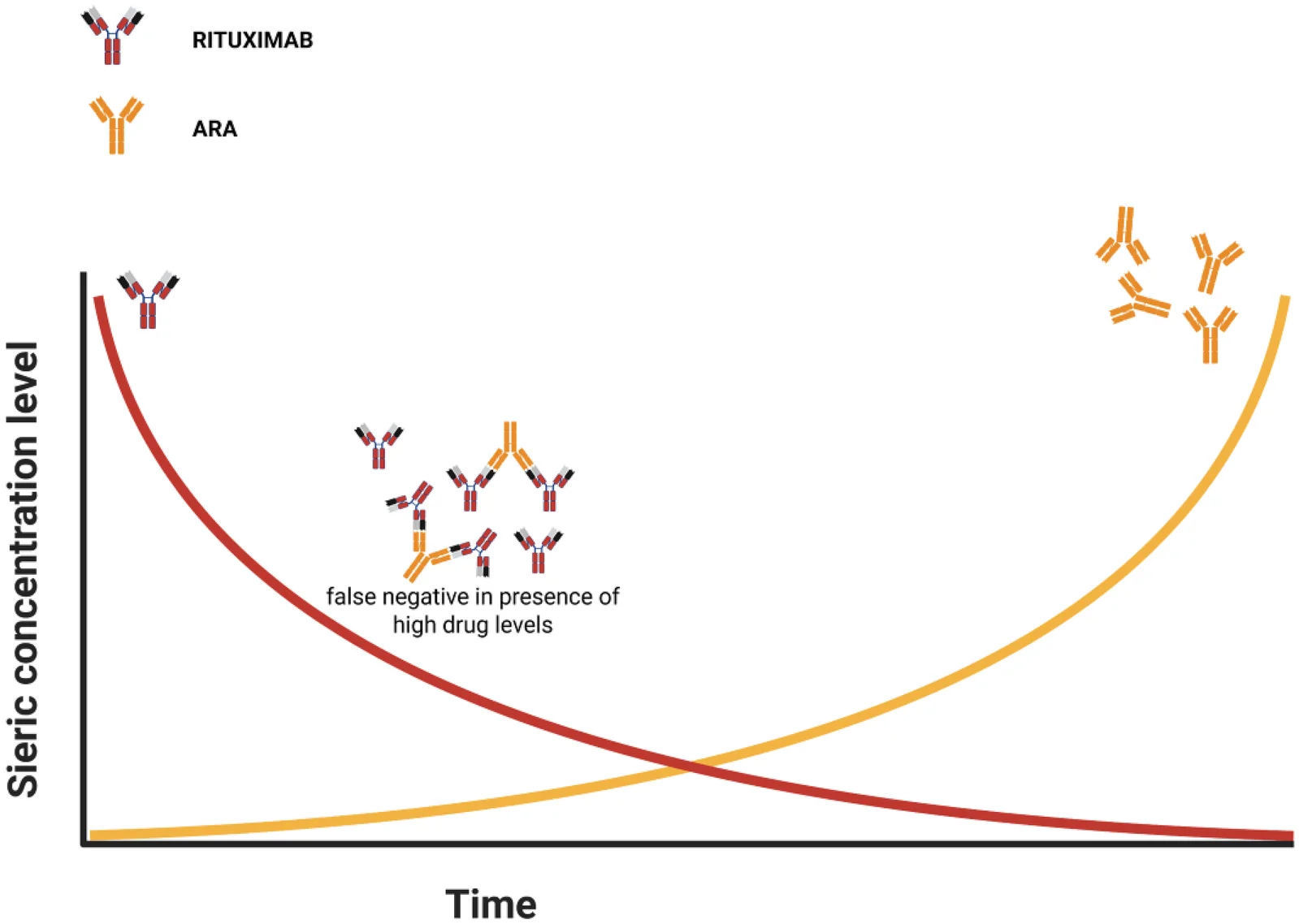
Schematic representation of the kinetics of rituximab and anti-rituximab antibodies (ADAs) over time. Following administration, rituximab serum levels progressively decline, while anti-rituximab antibodies increase over time. At high drug concentrations, ADA detection may be hindered due to drug interference, potentially leading to false-negative results. As rituximab levels decrease, free ADAs become detectable, resulting in an apparent increase in measured antibody levels.

##### 4.1.1.2 Electrochemiluminescence bridging assay

A newer and more sensitive method is electrochemiluminescence bridging assay (ECL), which has been adopted in different studies (11, 14, 18, 30, 40). It uses the same bridging principle, but with a double labeling of rituximab (biotin and ruthenium): the patient’s sample is incubated with chemiluminescent-labeled rituximab and rituximab immobilized on a solid phase (e.g., magnetic particles). In the presence of ADAs, a bridging complex is formed. After washing, the chemiluminescent reaction is activated, and the light emitted is proportional to the amount of ADAs present in a highly specific way and with less interference from free drug.

##### 4.1.1.3 RIA

Used in only one study (15), radioimmunoassay (RIA) offers great sensitivity but is rarely used today due to the need for specialized laboratories and the use of radioactive isotopes. It is also based on a bridging test: the patient’s serum is incubated with radioactively labeled rituximab (e.g., with iodine-125) and immobilized rituximab. If ADAs are present, they form a bridging complex between the two rituximab molecules. After washing to remove the unbound material, the residual radioactivity is measured and is proportional to the amount of ADAs present.

In summary, while ELISA remains a widely used method, ECL approaches are now preferable for increased reliability and sensitivity, especially in advanced clinical trials or in the management of patients with suspected refractoriness to treatment.

#### 4.1.2 Defining positivity cutoffs

The variability concerned both the units of measurement of the ADA titer [ng/mL, RU/mL (RU = reference units), U/mL, and AU/mL (AU = arbitrary units)] and the numerical cutoff. For studies using an ELISA testing method, the most commonly used cutoff was 5 ng/mL (19, 26–29). Clinical trials often define the cutoff based on control populations, as was the case in a study of SLE/AAV with ECL (baseline <2 AU/mL) (18).

#### 4.1.3 Detection timing

The interpretation of several studies is limited by variability in the timing of ADA measurements. In fact, samples for ADA detection in different studies were obtained at various, largely non-standardized time points after rituximab administration (**Table 2**).

Most studies involve a first test for the presence of ADAs 6 months after initial rituximab administration, often accompanied by a baseline sampling and one or more tests during the first year [e.g., pediatric SSNS (steroid-sensitive nephrotic syndrome) and SLE studies] (26). ADA levels gradually decline over time after rituximab discontinuation (14, 16, 70). Only a limited number of studies documented the persistence of ADAs in a few patients beyond 12 months (up to 36 months) after rituximab discontinuation (11, 16, 17, 54, 59). The Oxford SLE study reported the persistence of ADAs in 64% of cases for up to 36 months, indicating that a prolonged monitoring window may be useful in re-treated patients (11). Similarly, a recent study in patients with different nephropathies treated with rituximab (induction plus maintenance therapy) has also documented ADA positivity up to 36 months after rituximab treatment initiation (35).

ADA titers generally become undetectable after rituximab infusion in almost all patients and reappear with variable delay (>3 months in almost all patients) (16, 70). This finds a rationale because only the free fraction of ADAs, i.e., the one not bound to circulating rituximab, is detected with the bridging ELISA assay, and generally the circulating rituximab tends to disappear within the first 3–6 months of therapy (16, 71).

Most patients develop ADA in parallel with B-cell repopulation, and only less than 10% of patients were ADA-positive at a time when their relative B-cell count was 0% (35).

Measuring ADAs at baseline, before the first drug administration, is advisable to distinguish *de novo* ADAs from pre-existing antibodies, as in the case of patients previously treated with rituximab, requiring a new cycle of therapy (18). For 6-month maintenance regimens, testing during therapy may produce false negatives due to interference with the circulating antibody (16). ADA monitoring at months 9 and 12 after the last rituximab administration and before rituximab re-administration can identify almost 90% of patients with ADAs (16). The earlier the monitoring of ADAs after rituximab infusion, the higher the risk of false negatives.

### 4.2 Prevalence of anti-rituximab antibodies

The presence of anti-rituximab antibodies (ADAs) shows significant variability depending on the disease considered, the characteristics of the population studied, and the detection techniques used, with values ranging from almost zero percentages (59) up to over 50% (11).

In patients with SLE, including those with lupus nephritis, the mean prevalence of ADAs exceeds 40%, as evidenced by 11 studies published so far, with a percentage ranging from 15% to 74% (11, 12, 15: 18, 23, 27, 36–40). More recent data, such as those reported by Wincup et al. (11), demonstrate a positivity rate of around 38% already after the first cycle of treatment with rituximab. A longitudinal follow-up of 36 months also showed that 64.3% of patients maintain persistent ADA positivity over time, a condition associated with lower serum rituximab levels and an increased risk of clinical recurrence. Of particular note is that no significant differences in the prevalence of ADAs were observed between SLE patients with or without renal involvement (11).

In membranous nephropathy, the mean prevalence of ADAs is around 37.7% based on four available studies, with a percentage ranging from 23% to 47% (16, 19, 32, 33). These studies show a correlation between the presence of ADAs and the appearance of recurrences or treatment failure, underlining the importance of antibody monitoring in this disease.

In steroid-dependent or frequently relapsing pediatric nephrotic syndrome (SDNS/FRNS), the mean prevalence of ADAs is around 23%, with values ranging from 10.5% to 38% according to five studies (14, 26, 43–45). A further study reported a prevalence of 16.7% in children with nephrotic syndrome, confirming a notable frequency of antibody formation also in the pediatric setting (15). There are no data on adult nephrotic syndrome.

For antineutrophil cytoplasmic antibody (ANCA)-associated vasculitis (AAV), the prevalence of ADAs after rituximab treatment is lower and more variable, ranging from 0% to 25% (mean 12.5%) according to two main studies (18, 37). In Smith et al. (37), two patients with AAV and ADAs were both affected by microscopic polyangiitis (MPA) (37). In Faustini et al. (18), one patient with AAV tested positive for low-titer ADAs at baseline (prior to rituximab exposure) and tested negative after the therapy with rituximab. Moreover, this comparative study showed an absence of ADAs in patients with AAV after the first treatment with rituximab, whereas in patients with SLE the percentage reached 37.8%, suggesting important differences in the immunological profile between these autoimmune diseases (18).

In the study by Sentís et al. (4), the detection of ADAs was carried out in three distinct groups of patients suffering from kidney disease: hyperimmunized subjects in the pre-transplant desensitization phase, patients with acute humoral rejection after kidney transplantation, and, finally, patients with kidney disease in native kidneys. The latter group included heterogeneous conditions, including lupus nephritis, AAV, vesicoureteral reflux, focal segmental glomerulosclerosis, membranous nephropathy, polycystic kidney disease, neoplasms, and Alport syndrome. The overall prevalence of ADAs was low at 5.2% (one in 19 patients) but increased to 12.5% when only patients with native kidney diseases were considered, whereas it was absent in transplant patients (4). Similar results were observed in a transplanted pediatric cohort described by Zarkhin et al. (42), in which no ADA formation was detected, suggesting that post-transplant immunosuppression may reduce the risk of antibody response to rituximab (42).

A high prevalence of ADAs was reported in patients with autoimmune thrombotic thrombocytopenic purpura (TTP) by Vendramin et al., in which 42% of the subjects tested positive. However, it should be noted that ADAs have been investigated only in a subset of patients who had already developed acute or late reactions consistent with serum sickness, which may overestimate the true incidence in the general population of patients with autoimmune TTP. Despite this, early administration of rituximab in these patients has shown significant clinical efficacy, suggesting that the presence of ADAs does not necessarily negate its therapeutic benefits in this clinical setting (25).

In the context of autoimmune neurological disorders, the prevalence of ADAs was found to be, on average, 24.3% in neuromyelitis optica spectrum disorders (NMOSD) (49, 50), 37.6% in myasthenia gravis (48), 16.7% in primary progressive multiple sclerosis (PPMS) (13, 53), and 30.1% in relapsing–remitting multiple sclerosis (RRMS) (13, 51, 52). Therefore, in the context of multiple sclerosis, patients with RRMS appear to have a greater risk of becoming ADA-positive compared to those with PPMS.

Moreover, in the dermatological and rheumatology fields, notable percentages were observed: in Sjögren’s syndrome, a rate of 27% has been reported (41), while in pemphigus the prevalence varies between 18.2% and 31.0% (17, 54).

In rheumatoid arthritis (RA), the prevalence of ADAs is, on average, lower at approximately 9.3% according to data from 13 studies (22, 28, 30, 60–63, 65–69), but a single study with a small sample size (*n* = 31 patients) reported an unusually high frequency of 51.6% (64), suggesting the need for caution in interpreting these results.

In lymphoma, the prevalence of ADAs remains between 0% and 20.9% (8, 31, 55–58), with the highest values reported around 1 year of follow-up (72). This high variability appears to be related mainly to the timing of testing, in line with the findings reported in membranous nephropathy by Allinovi et al. (16), who documented the temporal trend of ADAs after rituximab infusion, with a peak at 9–12 months (16).

Finally, in the myalgic encephalopathy/chronic fatigue syndrome (ME/CFS) study, no ADA positivity was detected (59).

#### 4.2.1 Rituximab neutralization

The presence of ADAs does not necessarily imply a clinical impact. Since the immune system recognizes different parts (epitopes) of the infused mAbs, such as rituximab, only ADAs that recognize the CD20-binding region can exert a neutralizing activity (73–76). In relation to the polyclonal activity of ADAs, the neutralization test reports the percentage of neutralizing ADAs out of the total ADAs that a patient possesses (11, 19, 32, 74). The methods used to evaluate rituximab neutralization are varied and include cell-based assays (30), antibody-dependent but complement-independent cytotoxicity tests (19), and flow cytometry to analyze the blocking of the binding of the murine variable region of rituximab to CD20 on Epstein–Barr-immortalized B cells (54) as well as *in vitro* complement-dependent cytotoxicity assays (11, 19).

Neutralizing ADAs detected in a single patient’s serum are generally assessed by measuring the inhibition of rituximab-mediated cytotoxic activity (19, 24, 75). Therefore, the decrease in B-cell depletion is quantified by flow cytometry as a functional test of ADA-induced blockade (19) (**Figure 4**).

**Figure 4:**
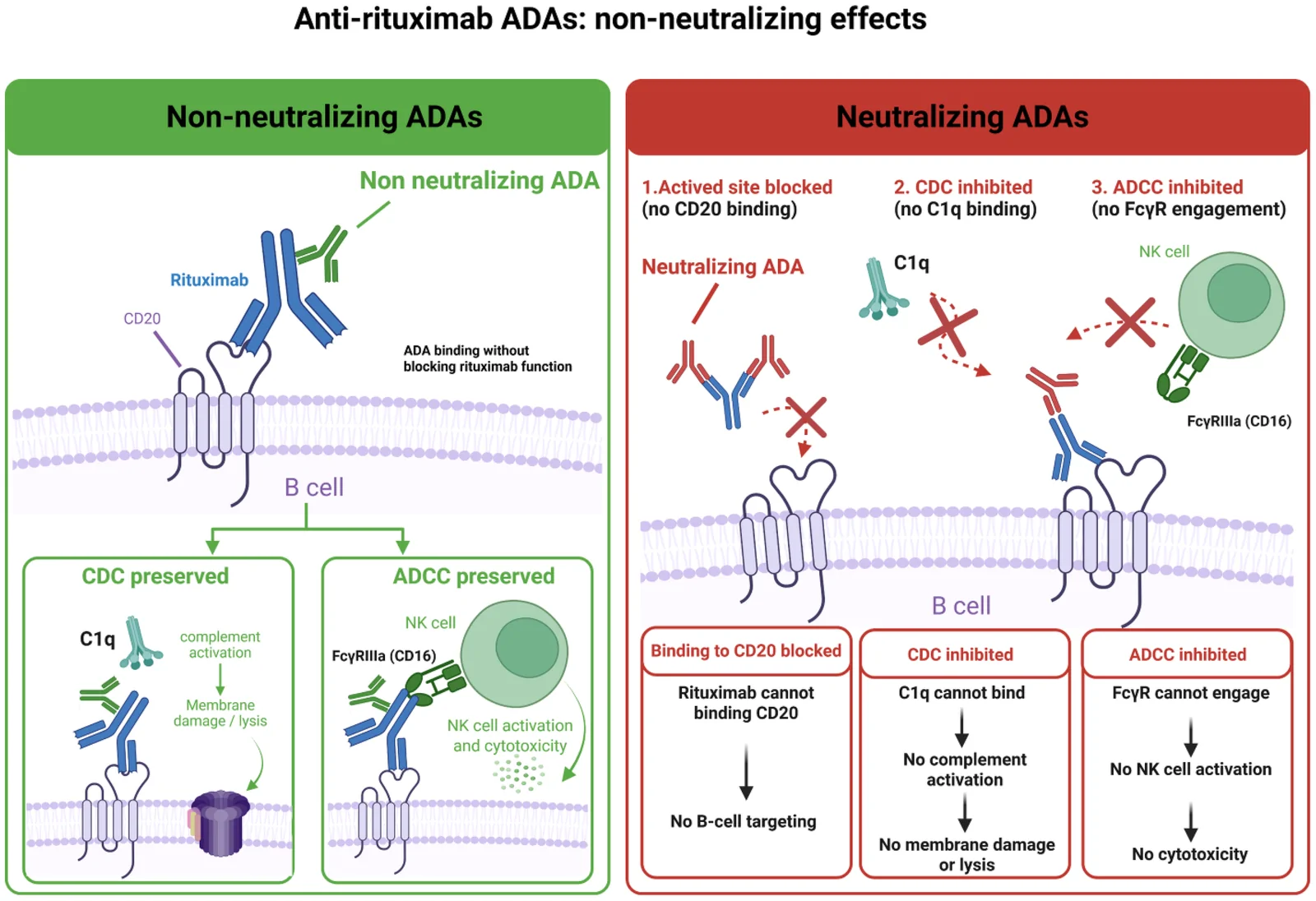
Non-neutralizing anti-drug antibodies (ADAs) bind rituximab without preventing its interaction with CD20, thereby preserving complement-dependent cytotoxicity (CDC) through C1q binding and antibody-dependent cellular cytotoxicity (ADCC) through FcγRIIIa-mediated natural killer cell activation. In contrast, neutralizing ADAs may inhibit rituximab activity by blocking its binding to CD20, preventing C1q recruitment and complement activation, and/or impairing Fcγ receptor engagement, ultimately reducing B-cell targeting, membrane damage, and immune-mediated cytotoxicity.

Testing their neutralizing activity is crucial to determine the capacity of ADAs to inhibit the therapeutic efficacy of rituximab. However, only a limited number of studies have directly evaluated this activity (11, 13, 19, 30, 49, 54, 57, 63, 65, 66, 68).

The analysis of sera collected from patients with ADAs capable of neutralizing rituximab shows considerable variability—for example, in patients with membranous nephropathy, a neutralizing activity in approximately 80% of cases is reported, suggesting a relevant role of ADAs in therapeutic failure (19). In a study on patients with SLE, Wincup et al. showed rituximab neutralization in 55.5% of ADA-positive patients, emphasizing how the presence of neutralizing ADAs may negatively affect serum rituximab levels and promote clinical relapses (11). Similarly, in membranous nephropathy, the presence of neutralizing ADAs has been correlated with the early recovery of B cells and an increased frequency of relapses, underlining the potential clinical impact of these antibodies (19). In contrast, studies performed in patients with rheumatoid arthritis (30, 63, 65, 66, 68), lymphoma (57), and pemphigus (54) reported an absence or low percentage of neutralizing activity despite the medium/high percentage of ADA-positive patients (**Table 2**), indicating that the presence of ADAs may not always result in a significant functional effect.

In summary, although the presence of ADAs is relatively frequent in patients treated with rituximab, their neutralizing capacity varies greatly among different diseases and patient characteristics.

#### 4.2.2 Potential role of anti-rituximab antibody titer

In children with frequently relapsing or steroid-dependent nephrotic syndrome, ADA titers negatively correlated with rituximab levels (47). Fujinaga et al. reported that all patients with high baseline ADA levels (≥100 ng/mL) showed shortened B-cell depletion and early disease relapse after subsequent rituximab treatment (14). Re-treatment with rituximab in previously ADA+ patients with subsequent undetectable ADA titer prolonged B-cell depletion, resulting in full recovery of rituximab efficacy (77).

Dunn et al. showed that, in multiple sclerosis, higher ADA titers were associated with incomplete B-cell depletion following rituximab administration, pointing to a possible neutralizing effect of these antibodies (13).

Conversely, in membranous nephropathy, two different studies suggested that the titer of ADAs does not correlate with clinical response or long-term outcomes (16, 35).

In addition, Wincup et al. reported a positive correlation between ADA titers and the occurrence of infusion-related reactions (40). In a different study on patients with different nephropathies, among six patients with unmeasurably high ADA levels (i.e., >100 ug/L), three developed infusion reactions and two responded well to further rituximab treatment (35).

No prospective study has validated a threshold of serum ADA levels to guide clinical decisions, particularly considering that the clinical impact of ADA may depend on their neutralizing capacity rather than absolute concentration alone (19).

However, in patients who develop ADA, it may be reasonable to re-administer rituximab once the ADA levels have tested negative or at least have decreased to <100 ng/mL or to consider switching to obinutuzumab in individuals with persistently high ADA levels.

### 4.3 Mechanisms of immunization against rituximab

The immunogenicity of rituximab is not solely determined by its origin (chimeric) but also by multiple factors related to drug composition, the patient characteristics, and the therapeutic strategy (**Figure 5**).

**Figure 5:**
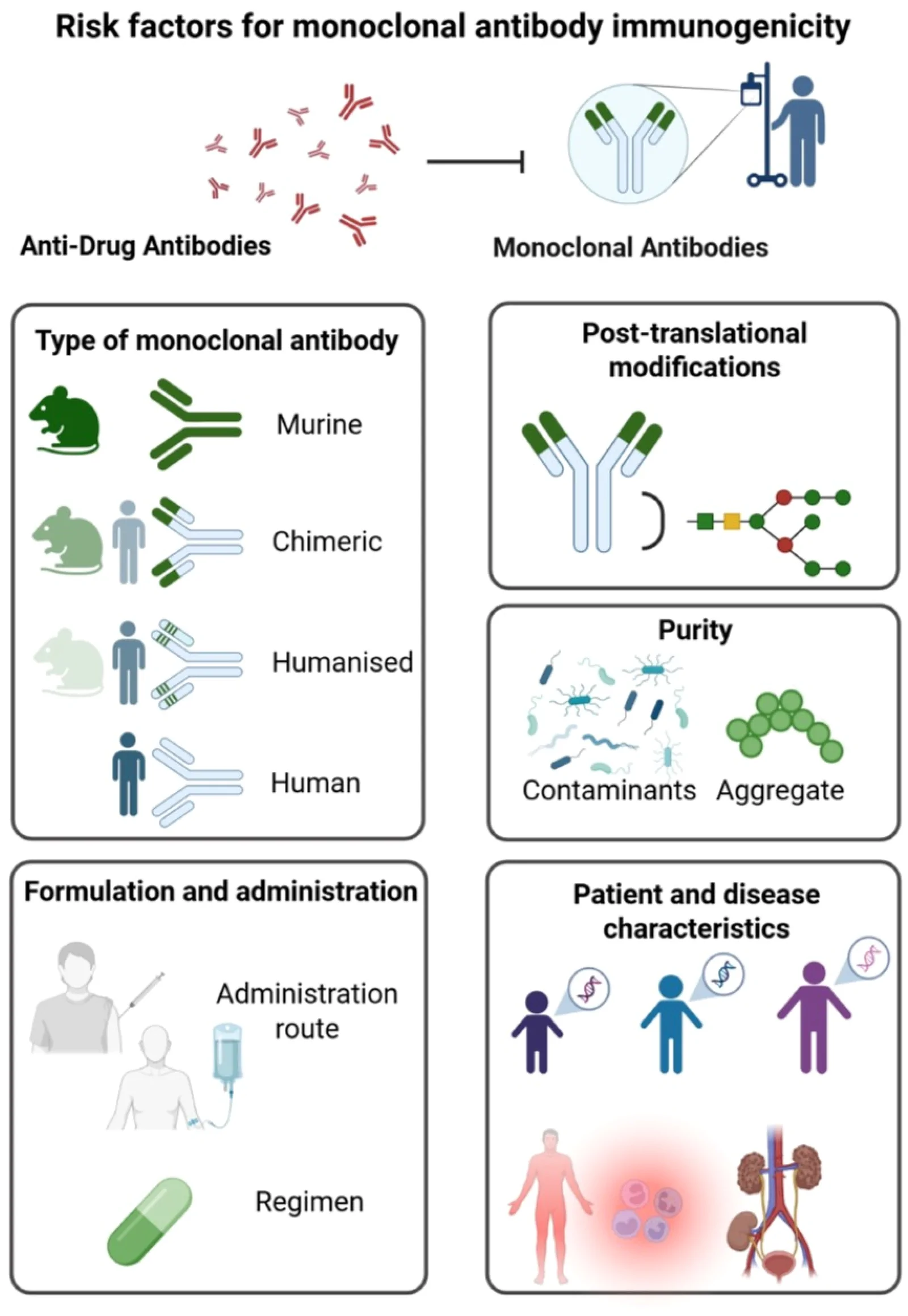
Risk factors influencing the immunogenicity of monoclonal antibodies (mAbs). The development of anti-drug antibodies (ADA) is a multi-factorial process influenced by drug-related and patient-related variables. The origin of the antibody affects its immunogenicity, with murine and chimeric proteins carrying a higher risk compared to humanized or fully human ones. Variations in glycosylation patterns can trigger immune recognition. The presence of microbial contaminants or protein aggregates increases the likelihood of an immune response. The way of administration (e.g., subcutaneous vs. intravenous) and the dosage regimen significantly impact on ADA formation. Genetic predisposition, the underlying disease state, and the patient’s immune status are critical determinants of the clinical response to mAbs.

#### 4.3.1 Antibody-structure-related factors

Due to their protein structure, chimeric antibodies are generally more immunogenic than humanized or fully human ones, though they can still elicit immune responses in some patients (78, 79). The murine portion, in particular the CDR (complementarity-determining regions) and FR (framework regions) of chimeric antibodies like rituximab, could be recognized as foreign by the human immune system, causing ADA production (80). Moreover, post-translational modifications—in particular, glycosylation patterns due to the cell-based platform used for mAbs production, such as Chinese ovarian cell lines—could be recognized as foreign antigens and trigger immune responses (81, 82). However, only a few data about the different immunogenicity of the glycosylation patterns of rituximab are available in the literature.

Moreover, misfolded or aggregated proteins may expose repetitive or novel epitopes that enhance immune activation and break tolerance, potentially leading to the formation of ADAs (83, 84). Regarding rituximab, due to its intrinsic immunogenicity caused by the chimeric nature, its aggregation had minimal effects on immune system activation compared to the monomeric drug (83).

#### 4.3.2 Formulation and administration factors

As reported by the Food and Drug Administration (FDA), rituximab excipients typical in drug formulations consist of polysorbate 80, salts, and water for injection. In particular, surfactants like polysorbate 80 could lead to the formation of micelles that may cause aggregation and enhance the immunogenicity of mAb (85).

Subcutaneous (SC) and intramuscular (IM) routes of administration are generally more immunogenic than the intravenous (IV) route, likely due to the prolonged exposure of the mAb to antigen-presenting cells very present at the peripheral level, particularly at the level of the draining regional lymph nodes. However, for rituximab, studies have shown no significant difference in immunogenicity between SC and IV routes (86).

High or repeated doses could induce immune tolerance and reduce ADA formation. Conversely, low residual drug levels have been associated with increased immunogenicity. Nonetheless, this relationship may vary depending on the disease and therapeutic context (87–89). In a cohort of relapsing–remitting multiple sclerosis patients (where 37% tested ADA positive) and in a cohort of progressive multiple sclerosis patients (where 26% tested ADA positive), ADA prevalence decreased with an increasing number of rituximab infusions (13).

#### 4.3.3 Patient-related factors

For rituximab, multiple host and disease factors modulate the risk of developing ADAs. Immune status is a primary determinant. In fact, profoundly immunocompromised patients, such as those with hematologic malignancies or recipients of solid-organ transplants, tend to mount fewer ADAs, whereas individuals with autoimmune disease show higher immunogenicity, consistent with immune overactivation and loss of tolerance (11, 56). In keeping with this, disease activity appears to matter: in SLE, higher baseline activity was associated with more frequent ADAs, and ADA-positive patients exhibited lower C3 levels in the first year after treatment. In membranous nephropathy, ADA positivity tracked with higher anti-PLA2R1 titers, epitope spreading, and increased IL-17A levels, all pointing to a more permissive immune milieu for anti-drug responses (11, 16, 18, 32). Conversely, ADA prevalence remains comparatively low in immunosuppressed hematologic cancers, again underscoring the role of background immune competence (56).

Genetic and ethnic factors may further shape risk. Although certain HLA variants are theoretically capable of enhancing peptide presentation from therapeutic antibodies (90, 91), no reproducible HLA association has been demonstrated for rituximab to date. By contrast, ethnicity has shown a signal in clinical cohorts: a cross-sectional study identified African ethnicity as a predictor of ADA development on rituximab (22).

Pharmacokinetic exposure likely also contributes. A larger volume of distribution or higher body weight can yield lower circulating drug concentrations, potentially compromising target coverage and facilitating immune recognition and ADA formation (16, 32, 92).

Vitamin D deficiency, given its immunomodulatory role (including effects on regulatory T cells), has likewise been linked to greater ADA risk in rituximab-treated patients (32).

Finally, age effects appear disease-specific rather than uniform across indications. In SLE, persistently ADA-positive patients were younger at SLE diagnosis (11), whereas in membranous nephropathy ADA-positive patients tended to be older (16).

### 4.4 Clinical significance of rituximab immunogenicity

#### 4.4.1 Anti-rituximab antibodies associate with lower circulating drug levels

In ADA-positive patients, rituximab plasma levels have been widely reported to be significantly lower than in ADA-negative patients. In particular, previous studies have shown this difference at 4 weeks (67) and at 2 months (26, 36) from the first administration. Two further studies confirmed this observation without specifying the precise timing of sampling (15, 47). These findings have been observed in patients with several autoimmune diseases, both with and without renal involvement, such as SLE, pediatric idiopathic nephrotic syndrome, and rheumatoid arthritis. In addition, Thurlings et al. documented the persistence of lower serum rituximab levels for up to 24 weeks after the first dose in ADA-positive patients (67). This underscores the importance of monitoring rituximab levels as a function of immunogenicity status. However, three studies in patients with membranous nephropathy, NMOSD, and pemphigus observed no significant differences in serum rituximab levels between ADA-positive and ADA-negative subjects (32, 49, 54).

#### 4.4.2 Anti-rituximab antibodies associate with lower B-cell (CD19+) depletion and earlier B-cell repopulation

Numerous studies have documented the reduced efficacy of rituximab in B-cell (CD19+) depletion in ADA-positive patients with SLE (12, 18, 36), multiple sclerosis (13), childhood-onset nephrotic syndrome (46, 47), and various pediatric immune-mediated diseases (15) compared to ADA-negative subjects. In addition, B-cell reconstitution has been observed to occur earlier in ADA-positive patients, with a significantly reduced duration of depletion in conditions such as membranous nephropathy (16, 19, 32, 93). Even in pediatric patients with SDNS, the duration of B-cell depletion is shorter in the presence of ADAs (14, 44). In parallel, in patients with NMOSD, an increased need for rituximab administrations to maintain effective B-cell depletion has been reported (50). However, some studies have not shown significant differences in B-cell depletion based on the presence of ADAs in conditions such as lymphoma, NMOSD, SLE, SDNS, and pemphigus (11, 31, 40, 43, 49, 54).

#### 4.4.3 Anti-rituximab antibodies associate with reduced clinical remission rate

One recent study was the first to demonstrate a significant association between ADAs and a reduced remission rate in 74 patients affected by membranous nephropathy (16). Previously, another study reported the same finding, but the result was not statistically significant, probably due to the relatively small sample (32). Another study described a less favorable response to rituximab in patients with ADAs affected by pemphigus but appeared to be limited by the small sample size (11 patients) (17). In patients with SLE, the ADA-positive patients still had a higher disease activity at 6 months of follow-up compared to ADA-negative patients (Mann–Whitney test, *p* = 0.004) (18). Another study, on children with frequently relapsing or steroid-dependent nephrotic syndrome, showed that ADA-positive patients needed higher cumulative rituximab treatments in order to maintain clinical remission (47). On the other side, various studies reported no association of ADAs with clinical response in patients affected by membranous nephropathy (19, 33), SLE (11, 38), RA (62, 67, 69), lymphoma (8, 55, 56), multiple sclerosis (13), myasthenia gravis (48), or pemphigus (54).

Since ADAs seem to reduce (and do not completely eliminate) the efficacy of rituximab, studies need to have a large sample size and a sufficiently long follow-up to demonstrate a statistically significant effect on the reduction of the clinical remission rate.

#### 4.4.4 Anti-rituximab antibodies associate with earlier and more frequent relapses

Several studies have shown that ADA positivity is related to a significant increase in the relapse rate in patients with membranous nephropathy (16, 19, 32, 93) and SLE (11). An analysis of patients with ANCA-associated vasculitis showed a similar trend but did not reach statistical significance, presumably due to sample size limitations (37). In pediatric SDNS, the relapse rate was significantly higher (46, 47), and the median relapse-free time after rituximab treatment was significantly shorter (14) in ADA-positive compared to ADA-negative patients. In contrast, other studies have found no significant differences in the relapse rate between ADA-positive and ADA-negative patients in settings such as SDNS, multiple sclerosis, NMOSD, and pemphigus (13, 26, 43, 49, 54).

#### 4.4.5 Anti-rituximab antibodies associate with hypersensitivity reactions

Biologicals are among the drugs that frequently cause hypersensitivity reactions (HSRs), particularly in ADA-positive patients (94). HSRs may occur during or within a few hours of drug administration (immediate HSRs) or within 14 days after the administration (delayed HSRs). HSRs in ADA-positive patients have been widely documented and include a broad spectrum of clinical manifestations ranging from urticaria–angioedema and rashes, chest or abdominal pain, diarrhea, dizziness to severe anaphylaxis in acute HSR, and fever, headache, arthralgia, purpura as well as serum sickness in delayed HSR (12, 14, 18, 22, 27, 28, 39, 41). Infusion reactions, occurring at the first exposure to rituximab, are generally caused by the massive release of cytokines from lysed cells and can be attenuated with steroid and antihistamine premedication as well as by a low rate of infusion (22, 95). On the other hand, in subsequent infusions, the presence of ADAs, both IgE and non-IgE (IgG and IgM) can cause both immediate hypersensitivity reactions (anaphylaxis) and delayed hypersensitivity reactions, such as serum sickness, due to the formation of immune complexes between rituximab and ADAs (96, 97). The incidence of HSRs in subsequent infusions is lower, ranging from 10% to 15% (22, 61). Specifically, ADAs belonging to the IgE isotype ADAs bind to mast cell receptors following sensitization, triggering massive degranulation with the release of mediators such as tryptase, histamine, prostaglandins, etc., potentially leading to a severe life-threatening allergic reaction. Conversely, IgG ADAs may activate immune cells, particularly basophils, resulting in the release of pro-inflammatory mediators. Additionally, both IgG and IgM ADAs may participate in the formation of immune complexes that can activate the classical complement pathway, thus contributing to the pathogenesis of HSRs (94).

Several studies have shown a significantly higher rate of HSRs in ADA-positive compared to ADA-negative patients with SDNS, SLE, or vasculitis (18, 27, 40, 44), while another one has reported similar results in a sample of patients with Sjögren’s syndrome, SLE, RA, or systemic sclerosis (22). It has also been observed that exposure to rituximab in patients with SLE or vasculitis is associated with a higher risk of HSRs in ADA-positive subjects, with an increase in serum sickness at the time of retreatment (18). In addition, recent multicenter research has identified specific clinical and laboratory parameters as significant predictors of infusion-related adverse reactions, emphasizing the importance of careful clinical monitoring in at-risk patients (98). Interestingly, among rituximab-related serious adverse events, HSRs are found to be the main category in which ADAs are detected (29). However, some studies have not found statistically significant differences in the rate of HSRs between ADA-positive and ADA-negative patients, probably due to sample size limitations or selection biases (14, 15, 25, 41, 46, 47). After all, also other studies in patients with membranous nephropathy, RA, lymphoma, multiple sclerosis, NMOSD, and pemphigus have shown no difference in HSR rate based on ADA status (13, 19, 30, 49, 52–54, 58, 66).

### 4.5 Strategies to reduce or bypass immunogenicity

#### 4.5.1 Optimization of dosing regimens

Several studies suggest that immunogenicity can be reduced by increasing drug exposure through high or repeated/more frequent dosing. However, this effect is not consistent across all diseases or treatments, and the evidence supporting these concepts is currently weak. Even considering that high doses may also increase the risk of toxicity, particularly through enhanced immunosuppression, an alternative strategy is to administer an inert but immunogenic version of the therapeutic antibody before treatment. This aims to induce immune tolerance without exposing the patient to the drug’s active toxicity (89).

#### 4.5.2 Co-administration of corticosteroids and/or immunosuppressive agents

Concomitant corticosteroid use in membranous nephropathy has shown conflicting results: in a study, steroids were shown to decrease ADA formation in patients treated with rituximab (34), whereas in another study they did not influence ADA formation (35). Immunosuppressive drugs could reduce the incidence of ADAs in patients exposed to biological agents—for example, concomitant methotrexate has been shown to decrease ADA formation in patients treated with anti-TNF therapies (99). However, in SLE, ADAs remain frequent despite immunosuppressive co-treatment (18). Given these inconsistencies and in the absence of a disease-specific indication, the routine addition of corticosteroids or immunosuppressant drugs to anti-CD20 therapy solely to reduce immunogenicity is not currently recommended.

#### 4.5.3 Drug desensitization

Drug desensitization (DD) is a procedure that allows for temporary clinical tolerance to a drug by administering gradually increasing small doses to complete the total therapeutic dose of the drug (11, 19, 100, 101). DD induces a change in mast cell reactivity with inhibition of mechanisms of their activation and degranulation (12, 100–102), as shown by the abolishment of specific skin test reactivity after each DD cycle. The mast cell unresponsiveness induced by DD is transient, and patients need to be re-desensitized each time they are exposed to the medication (103). However, experimental results provide evidence that, beyond the interference with mast cell function, the DD procedure may be able to modulate the adaptive immune response. In fact, *in vitro* monitoring of the biological-agent-specific effector cell response showed a decrease in drug-induced proliferation, while T-cell response to unrelated antigens resulted to be unmodified throughout the DD cycles (104).

The increase in circulating drug-specific Treg cells mainly producing IL-35 was shown during DD treatment, providing evidence that DD inhibits humoral and cellular anti-drug responses by increasing regulatory T cells and cytokines in an antigen-restricted manner. In fact, the increase in regulatory cytokines coincides with ADA decrease, pre-procedure skin testing negativization, and impairment of T-cell response (105).

The induction of IL-10, the classical regulatory cytokine, has also been described in successful DD (106, 107).

Rituximab desensitization is a safe, highly effective procedure for patients who have experienced HSRs to the medication, allowing them to continue treatment. It involves a 12-step or 16-step rapid protocol, typically over 5 to 6 h, using increasing, diluted doses to induce temporary tolerance.

However, desensitization protocols for monoclonal agents are followed in few centers, and most clinicians are unaware of the involved methods.

#### 4.5.4 Use of humanized or fully human monoclonal antibodies

Over time, humanized or fully human monoclonal antibodies have been developed. These have shown effectiveness in autoimmune and inflammatory diseases where chimeric antibodies have previously failed, particularly when ADA formation is involved. Anti-CD20 agents such as obinutuzumab, ofatumumab, and ocrelizumab offer increased *in vitro* and *in vivo* cytotoxicity compared to rituximab and may be effective in patients who develop ADAs (22, 24, 108). This may be due to the limited cross-reactivity between ADAs and the more humanized alternatives (19, 109). In addition, different studies support obinutuzumab as a possible effective and safe option for ADA-positive patients in both childhood-onset SDNS (70) and primary membranous nephropathy (21, 24, 93). Among children with SDNS and baseline ADA at obinutuzumab infusion, even those with ADA titers ≥100 ng/mL had no difference in B-cell depletion or relapse-free survival at 24 months (70).

Recently, a cohort of patients who developed anti-obinutuzumab antibodies was described (110). Although no information was available on neutralization capacity, the common development of anti-obinutuzumab antibodies in patients previously positive for ADA requires consideration of potential therapeutic strategies in these patients.

#### 4.5.5 Inducing immune tolerance and advances in antibody engineering

Recent advances in antibody engineering aim at minimizing immunogenicity by removing epitopes recognized as foreign by the immune system using bioinformatic and *in vitro* tools. Promising strategies include the use of regulatory T-cell therapies or fusion of tolerogenic peptides to monoclonal antibodies to induce specific immune tolerance. Additionally, reducing antibody aggregation and optimizing glycosylation profiles are key strategies to lower immunogenicity (111–113).

#### 4.5.6 Personalized treatment strategy

We propose an idea of a personalized treatment strategy based on ADA and drug level monitoring (**Figure 6**).

**Figure 6:**
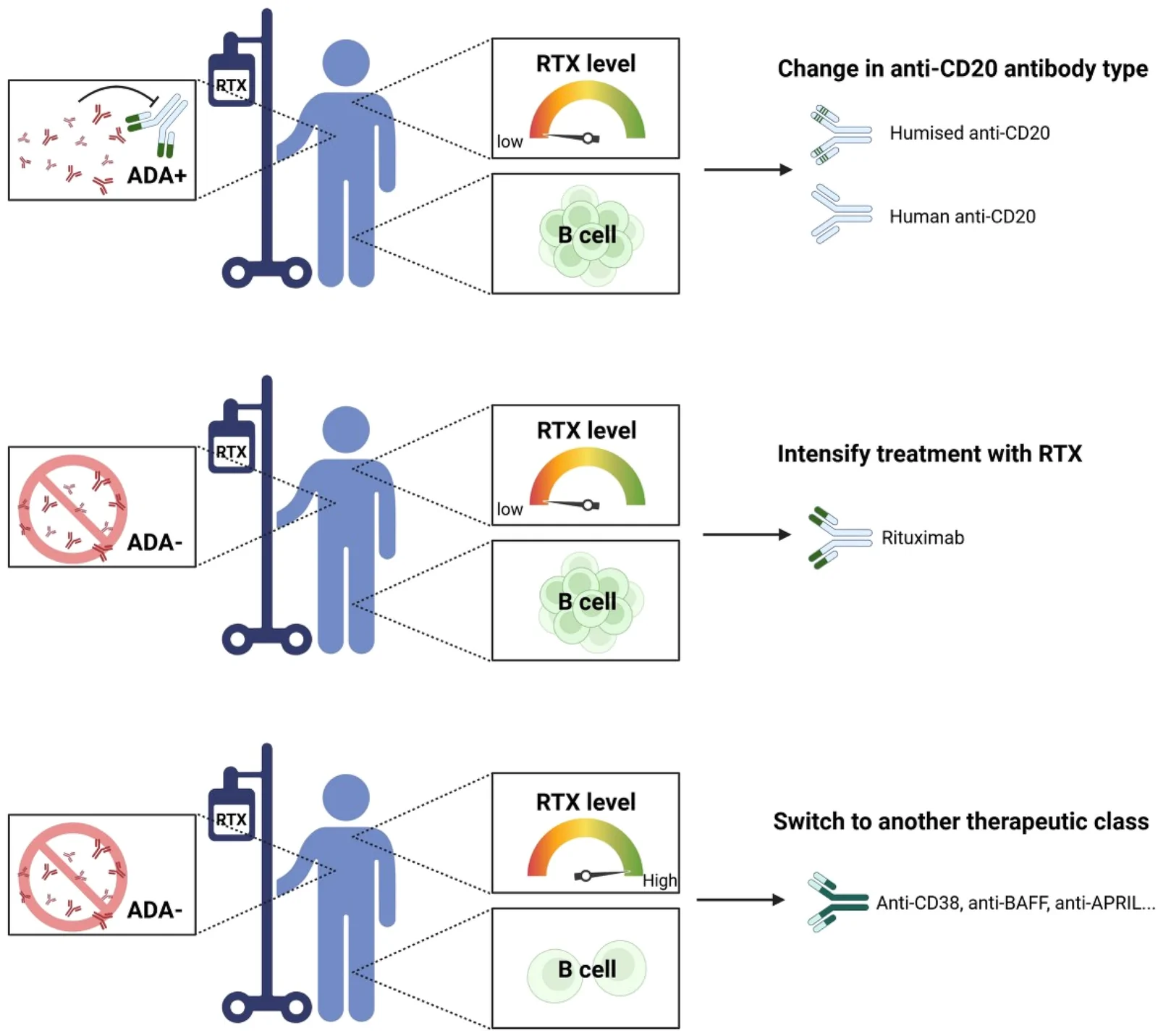
Possible therapeutic algorithms based on therapeutic drug monitoring of Rituximab (RTX). The clinical management of patients non-responding to RTX depends on the presence of anti-drug antibodies (ADA), serum drug levels, and the degree of B-cell depletion. In ADA-positive patients with low RTX levels and high B-cell counts, a switch to a less immunogenic anti-CD20 antibody (humanized or human) is recommended. In ADA-negative patients with low RTX levels and high B-cell counts, treatment intensification with RTX is indicated to achieve target therapeutic levels. In ADA-negative patients with high RTX levels but persistent B-cell activity, a switch to a different therapeutic class (e.g., anti-CD38, anti-BAFF, anti-APRIL) is required to overcome drug resistance.

Case no. 1: In ADA-positive patients, particularly in the presence of neutralizing antibodies and/or with high ADA titer, early B-cell repopulation and undetectable residual serum rituximab levels suggest treatment resistance related to immunization to the drug. The suggested management in this case is to consider switching to a humanized or fully human anti-CD20 monoclonal antibody.

Case no. 2: In ADA-negative patients, particularly in the presence of urinary rituximab loss, early B-cell repopulation and undetectable residual serum rituximab levels suggest treatment resistance related to drug underdosing. The suggested management in this case is to consider intensification of rituximab therapy (additional course, shorter intervals between courses, or dose escalation).

Case no. 3: In ADA-negative patients, detectable residual serum rituximab levels without early B-cell repopulation suggest primary treatment resistance. The suggested management in this case is to consider switching to another therapeutic class.

## 5 Discussion

This review summarizes the current evidence on ADA prevalence and their clinical significance in rituximab-treated patients, highlighting key aspects that have emerged from the most recent studies.

### 5.1 Test for ADA detection

The heterogeneity of the methods used to identify ADAs represents one of the main causes of the variability in the prevalence data reported. The different tests available, with varying sensitivity and specificity, could significantly influence the results and comparability between studies. This underlines the importance of standardizing diagnostic techniques and of cautiously interpreting results, especially in complex clinical settings. Overall, ELISA represents the most widely used test for ADA detection, with acceptable balance between costs and performance.

### 5.2 Prevalence and timing of ADA formation

The prevalence of ADAs varies greatly according to the disease and the population studied, with values that can exceed 40% in patients with SLE and membranous nephropathy and lower results in other conditions such as ANCA-associated vasculitis, rheumatoid arthritis, or hematological malignancies.

A relevant aspect concerns the timing of ADA formation: Numerous studies indicate that positivity could emerge already after the first cycle of rituximab, with follow-up documenting its persistence for months or years; even after approximately 15–21 months from the last administration, ADAs very often become negative. At the same time, ADAs’ disappearance and their reappearance after rituximab re-administration are documented phenomena, suggesting a complex immunological dynamic that requires further investigation. The more months passed since the last drug administration, the lower the free rituximab serum concentration, and the more likely it is to identify the presence of ADAs (if developed).

In clinical settings, particularly in conditions where maintenance therapy involves repeated dosing of rituximab every 6 months (such as SLE, vasculitis, and cryoglobulinemia), testing for ADAs during treatment may lead to false-negative results. For this reason, it is mandatory to combine ADA detection with the evaluation of rituximab serum levels in each serum sample.

### 5.3 Clinical implications of ADAs

The clinical impact of the presence of ADAs is mainly manifested in the reduction of serum rituximab levels and in the decreased efficacy of CD19+ B-cell depletion, with a consequent increase in the risk of recurrence or treatment failure in several diseases, especially in membranous nephropathy and SLE. A further important element concerns the impact on clinical remission: The presence of ADA not only has been shown to reduce the response to treatment but can also negatively affect the duration and quality of remission, making antibody monitoring a useful tool also for therapeutic management as well. Additionally, ADAs are associated with an increased risk of HSRs, which can range from mild manifestations to more serious events, both immediate and delayed. This requires close clinical monitoring during and after rituximab administration in ADA-positive patients.

The clinical relevance of ADA titer appears limited. Recent studies suggest that the mere presence or absence of antibodies may be more indicative than the quantitative measurement of antibody levels, the correlation of which with clinical outcomes remains uncertain.

Despite their potential clinical relevance, assays for the detection of neutralizing ADA are not routinely available and remain largely confined to research laboratories. Current approaches, including cell-based cytotoxicity assays, flow cytometric assessment of rituximab/CD20 binding inhibition, and CDC assays, are heterogeneous, technically demanding, and potentially influenced by experimental conditions. Moreover, the lack of standardized protocols, validated reference standards, and universally accepted positivity thresholds limits inter-laboratory comparability and currently prevents their use as stand-alone tools for therapeutic decision-making. Further prospective studies are required to develop standardized, reproducible, and clinically actionable neutralization assays.

### 5.4 Aspects to be explored in future studies

Future researchers are advisable to investigate the prevalence and the clinical impact of ADA in many nephropathies and other immune-mediated diseases, including large cohorts of patients.

Additionally, there is insufficient scientific evidence about the role of concomitant therapy with corticosteroids or other immunosuppressive drugs in reducing ADA development in rituximab-treated patients.

The available studies use different analytical platforms, positivity thresholds, sampling schedules, and reporting methods, making direct comparisons across studies and diseases difficult. The adoption of shared validation criteria, common terminology, and, ideally, international reference materials would therefore improve the reproducibility and clinical interpretation of results.

Prospective multicenter studies are also needed to clarify the potential role of therapeutic drug monitoring in patients receiving rituximab. Serial assessment of rituximab concentrations and ADA status should be combined with B-cell depletion and repopulation, disease activity, clinical relapse, and infusion-related reactions.

### 5.5 Limitations of the study

The findings of this review should be interpreted in light of several important limitations. The subjective nature of the narrative synthesis can introduce bias related to the lack of a standardized methodology, leading to conclusions with uncertain reliability. Notably, data from different diseases were compared despite substantial heterogeneity in immunopathogenesis, rituximab indications, dosing regimens, sampling timepoints, and ADA detection assays. These differences limited the comparability of the included studies and precluded a reliable quantitative synthesis. Consequently, the reported ADA prevalence ranges should be interpreted primarily within each disease-specific context.

The prevalence of ADAs is highly dependent on the sensitivity of the assay method used (e.g., ECL generally shows higher sensitivity than ELISA) and the timing of sampling relative to rituximab administration. Testing performed too early (e.g., within 3 months) may yield false-negative results due to interference from circulating rituximab, whereas sampling at 9–12 months post-infusion typically captures the peak ADA titer. These methodological differences contribute substantially to the wide prevalence ranges reported across studies and should be taken into account when interpreting the data.
